# Prenatal Diagnosis of Craniorachischisis Totalis Following an Initially Normal First-Trimester Ultrasound in a Woman With Two Previous Caesarean Births: A Case Report

**DOI:** 10.7759/cureus.110707

**Published:** 2026-06-11

**Authors:** Julius Mugisha, Stuart Turanzomwe, Ssewanyana Ernest, Charles Lugaaju, Esther C Atukunda, Mugyenyi R Godfrey

**Affiliations:** 1 Obstetrics and Gynaecology, Mbarara University of Science and Technology, Mbarara, UGA; 2 Obstetrics and Gynaecology, Kabale University School of Medicine, Kabale, UGA; 3 Medicine and Surgery, Mbarara Regional Referral Hospital, Mbarara, UGA; 4 Medicine, Divine Mercy Hospital - Father Bash Foundation, Mbarara, UGA; 5 Pharmacy, Mbarara University of Science and Technology, Mbarara, UGA

**Keywords:** anencephaly, craniorachischisis, neural tube defect, prenatal diagnosis, spina bifida

## Abstract

Craniorachischisis totalis is the most severe form of open neural tube defect and combines acrania/anencephaly with extensive open spinal dysraphism. It is rare, lethal, and usually identifiable prenatally, but diagnostic labeling may evolve across pregnancy as imaging findings become clearer.

We report the case of a 33-year-old multiparous woman, gravida 4 para 2+1, with two previous caesarean births, whose booking ultrasound at 8 weeks and 4 days of amenorrhea showed a single live intrauterine embryo with no documented structural abnormality. She later underwent targeted fetal anomaly scanning at 21 weeks, which revealed absence of the calvarium and parenchymal tissue above the orbits, with findings interpreted as anencephaly. A repeat assessment confirmed anencephaly with spina bifida. After multidisciplinary counselling and because of the combination of a lethal fetal anomaly and two prior caesarean scars, termination was done by hysterotomy at 25 weeks of gestational age. The woman delivered a male fetus weighing 659 grams with Apgar scores of 1 and 0 at 1 and 5 minutes, respectively; operative documentation described anencephaly with an open spine and spina bifida, findings most consistent with craniorachischisis. The mother had an uncomplicated postoperative course and remained clinically well at follow-up.

This case illustrates the evolution from an initially normal early pregnancy scan to a later lethal neural tube defect diagnosis, and it highlights the need for timely and targeted anomaly screening in resource-limited settings, careful phenotypic documentation, multidisciplinary counselling, and strong recurrence-prevention advice after an affected pregnancy.

## Introduction

Neural tube defects are among the most common serious congenital anomalies worldwide and remain a major cause of stillbirth, neonatal death, long-term disability, and pregnancy loss [1]. Recent global evidence suggests an average prevalence of about 2 per 1,000 births, corresponding to hundreds of thousands of affected pregnancies annually [1,2]. Across Africa, the burden of neural tube defects remains high. Recent systematic reviews and meta-analyses from the continent have shown a substantial pooled prevalence of neural tube defects, highlighting the need for prevention, surveillance, and improved maternal nutritional support [3]. A systematic review and meta-analysis from Eastern Africa estimated a pooled prevalence of 33.3 per 10,000 births, highlighting the clinical and public health importance of timely diagnosis and prevention in the region [4]. Ugandan data points in the same direction. Hospital-based birth-defect surveillance in Kampala, Uganda, has documented neural tube defects among births, confirming that these anomalies remain clinically relevant in Uganda even though phenotype-specific case reports remain limited [5].

Craniorachischisis is an especially severe and rare open neural tube defect in which both the cranial and spinal portions of the neural tube fail to close [6]. In its total form, it combines anencephaly with extensive open spina bifida and is considered uniformly lethal [6].

Prenatal ultrasound is central to diagnosis [7]. Neural tube defects can often be identified antenatally, and anencephaly-related phenotypes may be detected in the late first trimester or early second trimester when skull and cranial tissue abnormalities become evident [8].

To prevent neural tube defects, WHO recommends daily folic acid supplementation from the time a woman begins trying to conceive until 12 weeks of gestation, because neural tube closure occurs before many women recognize pregnancy [9]. Women with a previously affected pregnancy require strong recurrence-prevention strategies in subsequent pregnancies [10,11].

We report a Ugandan case in which an apparently normal first-trimester scan was followed by a second-trimester diagnosis of anencephaly, with post-delivery findings supporting craniorachischisis, and we discuss the diagnostic, counselling, and recurrence-prevention implications.

## Case presentation

A 33-year-old pregnant woman, gravida 4 para 2+1 with two previous caesarean births, presented for booking antenatal care at 8 weeks and 4 days of gestation. Her vital signs were normal, and the recorded concerns at that visit were cough and flu symptoms rather than pregnancy-related complaints. The laboratory assessment revealed blood group AB RhD positive, a negative HIV screening test, and a negative treponema pallidum hemagglutination assay (TPHA) result, while urine testing and full blood count were recorded as normal (Table 1). Obstetric ultrasound at that visit showed a single live intrauterine embryo with cardiac activity, a normal yolk sac, no subchorionic hemorrhage, no adnexal mass, no free pelvic fluid, and a crown-rump length of 1.95 cm, corresponding to 8 weeks and 4 days. The cervix was closed and of normal length, and no structural fetal anomaly was documented at that time. She was given 5 mg of folic acid daily for 40 days. Two weeks later, she returned with complaints of cough and flu symptoms and was treated as having an upper respiratory tract infection in a first-trimester pregnancy. She, however, had no obstetric complications.

**Table 1:** Laboratory tests done during antenatal care screening TPHA: treponema pallidum hemagglutination assay; MCV: mean corpuscular volume; MCH: mean corpuscular hemoglobin; MCHC: mean corpuscular hemoglobin concentration; RDW-CV: red blood cell distribution width - coefficient of variation; PDW: platelet distribution width; MPV: mean platelet volume

Test	Result	Reference Range
Blood Group	AB Rhesus D Positive	—
HIV 1/2 Antibody Test	Negative	Negative
TPHA (Syphilis test)	Negative	Negative
Random Blood Sugar (RBS)	8.2 mmol/L	4.2 – 7.2 mmol/L
Complete Blood count		
WBC	10.33 ×10³/µL	3.1 – 9.1
Neutrophils #	8.03 ×10³/µL	2.5 – 7.5
Neutrophils %	77.8%	45 – 75%
Lymphocytes #	1.84 ×10³/µL	1.3 – 4.0
Lymphocytes %	17.8%	25 – 55%
Monocytes #	0.30 ×10³/µL	0.12 – 1.20
Monocytes %	2.9%	4.5 – 13%
Eosinophils #	0.14 ×10³/µL	0.02 – 0.50
Basophils #	0.02 ×10³/µL	0.00 – 0.10
Red Blood Cell (RBC)	4.40 ×10⁶/µL	3.9 – 6.0
Hemoglobin (HGB)	12.8 g/dL	12.2 – 16.8
Hematocrit (HCT)	37.4%	35.0 – 50.8
MCV	84.9 fL	76 – 96
MCH	29.1 pg	27 – 32
MCHC	34.3 g/dL	32 – 36
RDW-CV	13.8%	11 – 16
Platelets (PLT)	134 ×10³/µL	150 – 450
PDW	17.3 fL	9 – 17
MPV	10.3 fL	9 – 13
Urinalysis		
Urine Appearance	Pale yellow and clear	—
Urine Glucose	Negative	Negative
Urine Ketones	Negative	Negative
Specific Gravity (SG)	1.025	1.00 – 1.030
Urine Blood	Negative	Negative
Urine pH	6.0	5.0 – 9.0
Urine Protein	Negative	Negative
Nitrites	Negative	Negative
Leucocytes (Urine)	Negative	Negative
Urine Microscopy	Normal	—

Later in the second trimester, she had targeted imaging for foetal anomalies (TIFFA) done at 21 weeks of gestation, which revealed a single live intrauterine fetus with normal cardiac activity, gross movements, tone, intact anterior abdominal wall, a three-vessel cord, a posterior placenta, adequate amniotic fluid, and a closed cervix. However, the scan also showed the absence of the fetal calvarium and the absence of parenchymal tissue above the orbits, and the findings were interpreted as being suggestive of anencephaly. Another ultrasound was done from another facility and confirmed anencephaly with spina bifida. Because of the fetal anomaly and the maternal history of two previous caesarean births, which is a contraindication for vaginal delivery as per local guidelines, counselling about the prognosis and pregnancy termination by caesarean delivery at 24 to 26 weeks was done by a multidisciplinary team comprising an obstetrician, a pediatrician, and a social worker.

**Figure 1:**
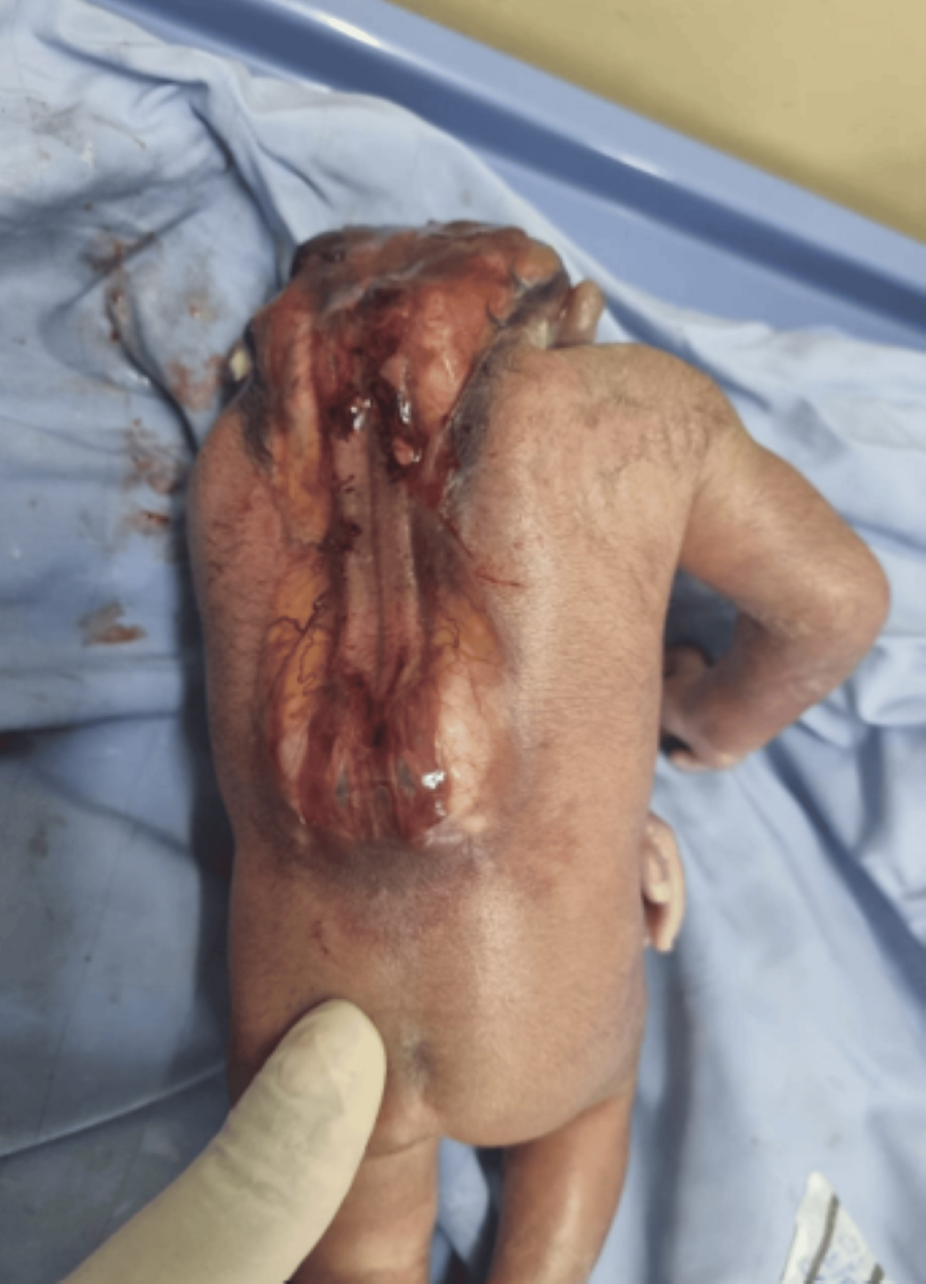
Back view of the newborn showing extensive spinal defect

**Figure 2:**
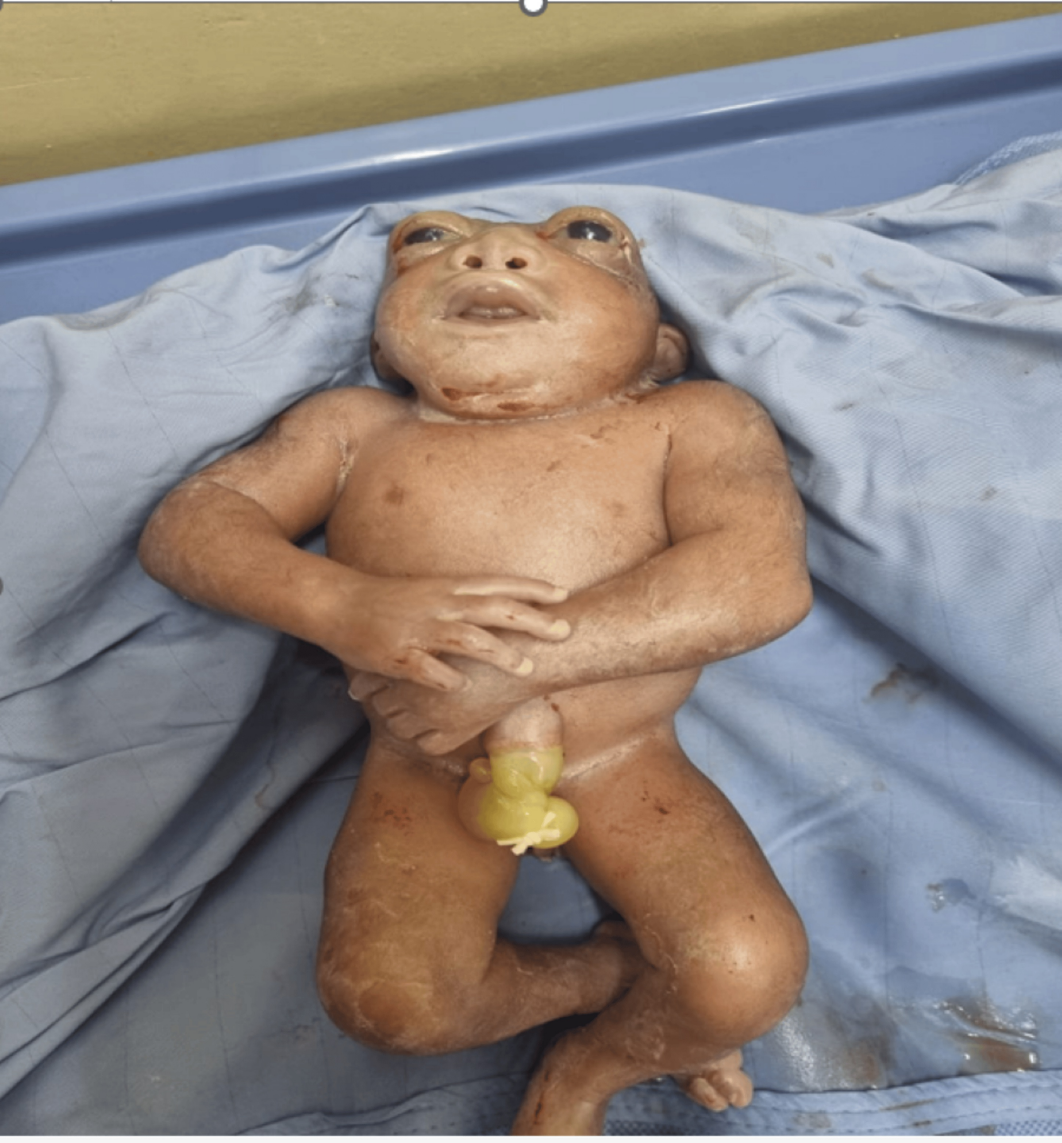
Anterior view of the newborn showing absent calvarium and "frog-eye" appearance

**Figure 3:**
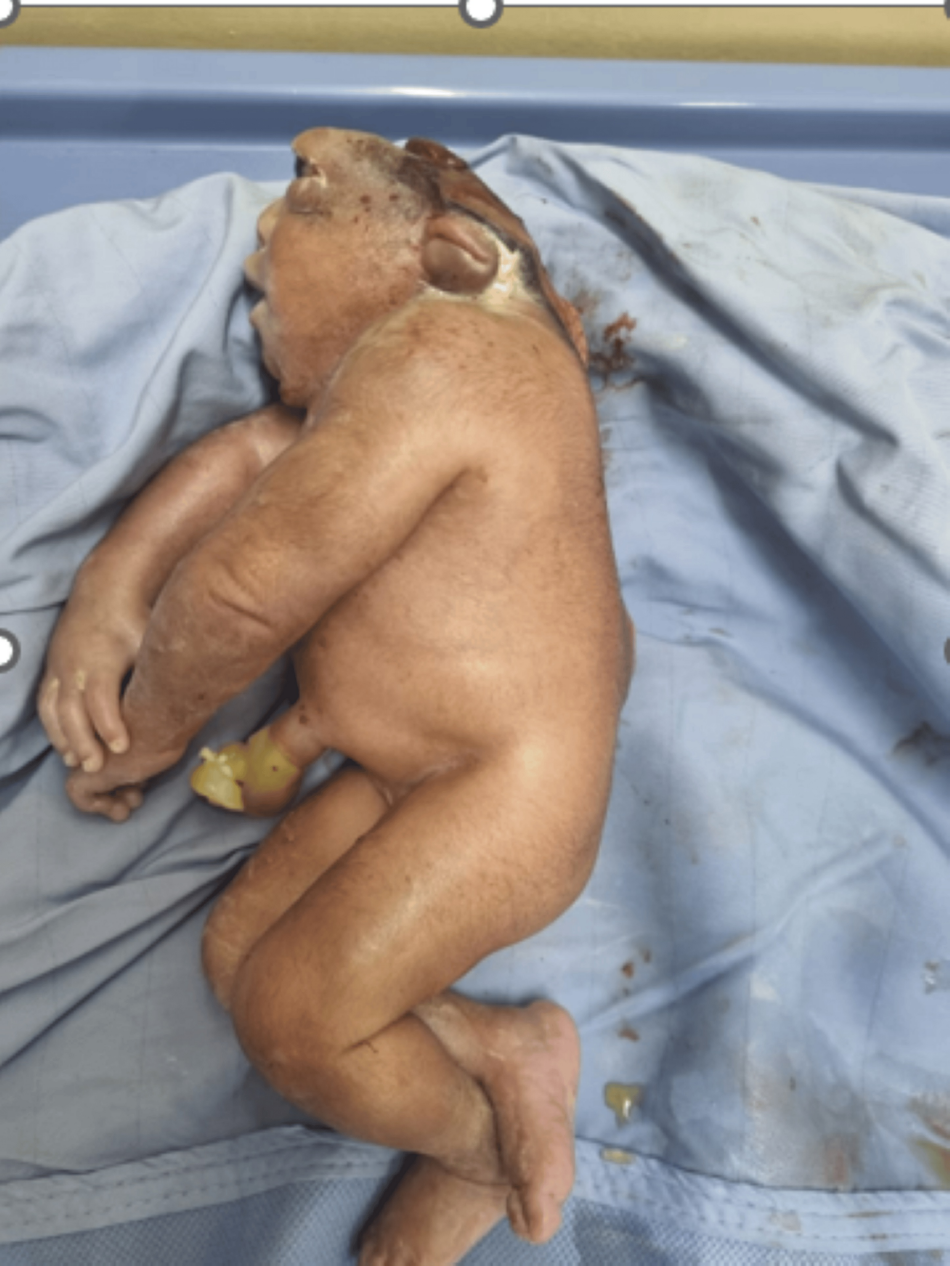
Lateral view of the newborn

At 25 weeks of gestation, she underwent elective hysterotomy under spinal anesthesia as she had two previous caesarean sections, polyhydramnios, and anencephaly, and local guidelines don’t allow induction of labour in the presence of two or more previous caesarean section scars. A male fetus weighing 659 g was delivered with Apgar scores of 1 at 1 minute and 0 at 5 minutes. No resuscitation was done in view of the gross anomalies seen. The baby had anencephaly with an open spine and spina bifida, which, taken together, is most consistent with craniorachischisis totalis rather than isolated anencephaly (Figures 1-3) [6]. Postoperatively, the mother remained stable and was discharged with no new complaints. Postnatal follow-up was done at 5 weeks post-delivery. She had no complaints and received a contraceptive implant (Implanon; Organon & Co., Jersey City, USA).

## Discussion

This case highlights a severe form of open neural tube defect managed in a rural Ugandan setting. It also shows the diagnostic evolution from an apparently normal first-trimester scan to a later anomaly diagnosis. Thirdly, it highlights the difference between antenatal labeling as anencephaly and final phenotypic classification as craniorachischisis totalis after operative documentation of an open spine [6].

The final diagnosis in this case is best interpreted clinically as craniorachischisis, most likely craniorachischisis totalis, despite the absence of genetic and pathological confirmation, because the recorded delivery findings combined anencephaly with an open spine/spina bifida. Craniorachischisis totalis, by definition, involves failure of closure of both cranial and spinal portions of the neural tube, whereas isolated anencephaly refers only to the cranial component [6,8].

The early normal ultrasound at 8 weeks does not necessarily contradict the later diagnosis. Neural tube closure occurs very early in embryogenesis, but recognition of phenotype on ultrasound depends on gestational age, image quality, and the specific anomaly sought. Reviews of prenatal diagnosis note that anencephaly and related cranial defects are often identified in the late first trimester or early second trimester scan, like the nuchal translucency scan, rather than at a very early booking scan [8,12,13].

The initial targeted scan described the spine as appearing normal, yet repeat imaging identified spina bifida, which was further confirmed post-operatively. This discrepancy may reflect limitations of fetal position, gestational timing, operator dependence/interobserver bias due to level of training/experience, quality of machine, which influences image resolution, or documentation practices rather than true absence of spinal dysraphism [13,14].

The management course was also shaped by maternal obstetric history. Because the patient had two previous caesarean scars, the team chose termination by hysterotomy rather than induction, after multidisciplinary discussion and counselling. In this respect, the case is not only about fetal diagnosis but also about tailoring management of lethal anomaly to maternal surgical history and local procedural judgment.

The case reinforces the continuing need for stronger preconception prevention. Folic acid remains the best-established primary preventive intervention for folate-sensitive neural tube defects [9]. The Medical Research Council (MRC) Vitamin Study showed that periconceptional folic acid substantially reduces recurrence risk, and the Hungarian randomized trial showed a reduction in first occurrence with periconceptional vitamin supplementation [15]. WHO and the Ministry of Health, Uganda recommend daily folic acid supplementation from the time a woman intends to conceive, preferably 90 days before, and continued until 12 weeks’ gestation, since neural tube closure occurs before many women recognize pregnancy [9]. In this case, folic acid was prescribed at the booking antenatal care visit, which was after the relevant embryologic window for primary prevention, highlighting the need for continued preconception education and supplementation. This timing issue is especially important in African settings, where the burden of neural tube defects remains high, and surveillance data suggest substantial room for better preconception care and prevention [4,16]. The practical implication is that prevention must move upstream from antenatal folate prescription to preconception counselling, supplementation, and, where possible, food fortification [17].

The case also highlights post-event counselling needs. Women with a previously affected pregnancy are commonly considered at elevated recurrence risk and are generally advised to use higher-dose folic acid before a subsequent conception [18]. The documented follow-up in this case included postoperative review, family planning, recurrence counselling, and preconception planning; however, genetic evaluation was not undertaken.

However, this report had some important limitations. There was no record of maternal diabetes status, anticonvulsant exposure, detailed nutritional history, body mass index interpretation, family history of congenital anomalies, consanguinity, socioeconomic context, and absence of pathology/genetic confirmation, especially in this local setting. These omissions limit inference about etiology and reduce phenotypic certainty.

## Conclusions

This case describes a pregnancy with an initially reassuring early ultrasound that later evolved into antenatal diagnosis of a severe neural tube defect - anencephaly - with postpartum findings most consistent with craniorachischisis totalis. The case underscores the value of formal anomaly scanning, careful phenotypic documentation, multidisciplinary counselling, individualized obstetric planning in women with previous caesarean scars, and explicit preconception folate-based recurrence prevention after an affected pregnancy.
